# Can the Dark Side of Employee Innovative Behavior Be Mitigated by Frequency of Supervisor Interaction? Analyzing the Moderated Mediation of Envy and Ostracism Through Frequency of Supervisor Interaction

**DOI:** 10.3390/bs15111463

**Published:** 2025-10-28

**Authors:** Eunmi Jang, Heeyeob Kang

**Affiliations:** 1 Baird Liberal Arts College, Soongsil University, Seoul 06978, Republic of Korea; 2 Department of Physical Education, Chosun University, Gwangju 61452, Republic of Korea

**Keywords:** innovative behavior, envy, workplace ostracism, frequency of supervisor interaction

## Abstract

While innovative behavior is essential for organizational success, recent studies have highlighted its potential dark side, namely triggering envy and ostracism among coworkers. However, we propose that these negative outcomes are contingent on the organizational context, particularly on the frequency of supervisor interactions. Using multi-wave data from 392 South Korean employees, we demonstrate that the frequency of supervisor interaction fundamentally alters the social impact of innovative behavior. Our findings reveal a striking pattern: When frequency of supervisor interaction is low, innovative behavior indeed triggers the predicted dark side—increasing ostracism through heightened envy. However, when frequency of supervisor interaction is high, this relationship reverses—innovative behavior reduces ostracism by suppressing envy. This moderated mediation effect suggests that the dark side of innovation is not inherent but context-depenent. We theorize that high-frequency supervisor interaction transforms innovative behavior from a competitive threat to a collective asset. These findings challenge deterministic views of creativity’s social costs and highlight the critical role of leadership in shaping how innovation is interpreted and responded to within organizations.

## 1. Introduction

Innovative employee behavior is essential for an organization’s sustainable competitive advantage (Amabile, 1996). Simultaneously, researchers have increasingly noted that innovation may incur interpersonal costs, often referred to as the “dark side of creativity.” For instance, employees who generate new ideas may unintentionally trigger envy, resentment, or even ostracism among colleagues who perceive them as deviating from group norms or threatening established hierarchies (Breidenthal et al., 2020; Mao et al., 2021). Such findings highlight that innovation is not uniformly celebrated but can carry relational risks alongside its performance benefits.

However, the empirical evidence regarding these interpersonal consequences remains unclear. Some studies have documented exclusionary responses to innovators, suggesting that highly creative employees may be targets of envy or passive resistance. In contrast, other studies report null or even positive effects, showing that innovation can promote collegial respect, knowledge sharing, and collective pride when embedded in supportive contexts (Perry-Smith, 2006; Černe et al., 2014). This inconsistency suggests that the consequences of innovative behavior are neither universal nor automatic. Rather, they are shaped by how coworkers interpret innovation in their social environments. Uncovering the emotional mechanisms and contextual conditions that determine when innovation provokes ostracism and fosters integration is a critical question for both theory and practice.

According to social comparison theory (Festinger, 1954), employees who demonstrate superior performance often become the target of upward comparison, which can elicit envy among colleagues. However, the outcomes of these comparisons are not predetermined. Cohen-Charash and Mueller (2007) showed that superior performance triggers envy when perceived as a threat, but can also foster admiration and learning opportunities when framed as a source of inspiration and growth. This suggests that whether innovative behavior provokes or suppresses envy depends critically on how it is interpreted within the social context.

Recent studies support this conditional view. Breidenthal et al. (2020) and Mao et al. (2021) found that coworkers’ envy mediated the link between employee creativity and ostracism, demonstrating a dark side mechanism whereby innovation provokes exclusion. At the same time, other researchers have noted that envy has dual forms: malicious envy fosters ostracism and resistance, but benign envy can stimulate motivation and innovative behavior (Tai et al., 2012). Studies have also shown that when innovation is embedded in supportive climates or knowledge-sharing cultures, it can reduce interpersonal tension and promote collective pride (Černe et al., 2014; Perry-Smith, 2006). These findings emphasize that envy is not an automatic, uniformly negative reaction but rather a contingent emotional mechanism shaped by interpretive cues.

We argue that supervisors play a pivotal role in the structuring of interpretive cues. As organizational sense givers (Maitlis, 2005), supervisors define how innovative behavior should be understood—either as individual differentiation or as a collective contribution to group success. When supervisors highlight innovative behavior as an individual’s distinctive accomplishment, it may sharpen upward comparisons, increase coworker envy, and heighten the risk of ostracism. Conversely, when supervisors frame innovation as aligned with team goals and organizational benefits, coworkers are more likely to interpret such behavior positively, reducing envy and thereby reducing the likelihood of exclusion. Leader support has been found to buffer the negative social effects of innovation: for instance, Breidenthal et al. (2020) showed that leaders’ public recognition mitigated envy-driven ostracism, while other studies demonstrate that supportive leader behaviors enhance the positive spillover of innovation into employee wellbeing (Wang et al., 2022).

The current study was conducted in South Korea, where collectivistic and relationship-oriented cultural norms strongly influence the interpretation of innovative behavior. In such contexts, individual achievements may be perceived not only as personal success but also as threats to group harmony and relational stability. Prior research shows that high performers and creative employees often experience envy from coworkers, which can result in ostracism (Kim & Glomb, 2014; Mao et al., 2021). Importantly, in collectivistic cultures where face-saving and hierarchy are salient, the frequency of supervisor interaction is a critical moderating factor. Supervisors act as sense givers who can frame innovation as a collective contribution rather than individual self-promotion, thereby legitimizing behavior and reducing perceptions of threat (Lu et al., 2022). Empirical studies further suggest that leaders’ support can buffer the dark side of innovation while enhancing employee wellbeing (Wang et al., 2022). Accordingly, our finding that innovative behavior in the Korean workplace can reduce envy and ostracism should be interpreted within this specific cultural framework. This offers important insights into how collectivistic values and supervisor–subordinate interactions jointly shape the social consequences of innovation compared to in Western individualistic contexts.

Building on this logic, this study had three objectives. First, it examined the relationship between innovative behavior and ostracism in Korea’s relationship-oriented workplace context, considering the possibility—unlike much of the Western literature—that innovative behavior can reduce ostracism when perceived as a group contribution. Second, it investigated envy as the mediating mechanism that links innovative behavior to ostracism, positioning envy as the emotional black box that clarifies how innovation is translated into exclusionary outcomes. Third, it analyzed how the frequency of supervisor interaction moderates this process, testing whether high-quality supervisor interaction strengthens the positive effects of innovative behavior (i.e., reduced ostracism via decreased envy).

This study sought to empirically demonstrate through a longitudinal survey of 392 South Korean employees that the dark side of innovation is a conditional phenomenon rather than a universal one. By highlighting the mediating role of envy and the moderating role of the frequency of supervisor interaction, we aim to contribute nuanced theoretical insights into when innovation provokes ostracism and fosters cohesion, while also offering practical guidance for organizations to encourage innovation without jeopardizing relational harmony.

## 2. Theory and Hypotheses

### 2.1. Reexamining the Relationship Between Innovative Behavior and Workplace Ostracism

Traditional perspectives on innovative behavior have primarily emphasized the potentially negative consequences of interpersonal relationships. Based on social comparison theory (Festinger, 1954), researchers have argued that innovative employees become targets of upward comparison, causing relative deprivation among colleagues. Mao et al. (2021) empirically demonstrated that innovative employees are perceived as monopolizing scarce organizational resources such as recognition, rewards, and promotion opportunities, thus triggering hostility from colleagues. Similarly, Breidenthal et al. (2020) provide empirical evidence that creative performance stimulates colleague envy, leading to exclusionary behavior.

From this perspective, innovative behavior is interpreted as inherently competitive and threatening. Innovative individuals are perceived as entities who disrupt the status quo, challenge existing power structures and status systems, and diminish the relative value of other organizational members (Janssen, 2003). However, we propose that these negative outcomes are not universal but rather vary according to the organizational and cultural contexts in which innovative behavior occurs.

We present three mechanisms by which innovative behavior can reduce, rather than increase, ostracism. First, innovation creates positive spillover effects that benefit the entire organization. Perry-Smith and Mannucci (2017) argued that innovation creates benefits for the entire organization beyond individual achievement. When employees’ innovative ideas improve team processes, introduce valuable knowledge and skills, and enhance organizational competitiveness, all members experience indirect benefits. From this perspective, innovative behavior represents a positive-sum game rather than a zero-sum game.

Second, cultural context plays an important role in shaping the interpretation of innovative behavior. According to Hofstede’s (1980) cultural dimension theory, collectivistic cultures tend to interpret individual achievements as group achievements. In relationship-oriented cultures like Korea, individual success is understood as “our” success, innovation enhances group pride and honor, and harmony and cooperation are valued over competition. Therefore, innovative employees are more likely to be perceived as valuable group members than as threatening competitors.

Third, from the perspective of social learning theory (Bandura, 1977), innovative behavior provides important learning opportunities for other organizational members. By observing colleagues’ innovation, employees can acquire new thinking patterns and problem-solving approaches, gain motivation to develop their creative potential, and confirm that innovation is valuable and achievable within the organization. This positions innovative employees as role models, rather than targets for exclusion.

Integrating these theoretical perspectives, we propose that innovative behavior does not inevitably lead to ostracism. Particularly in the context of Korean organizations, where innovation is interpreted as enhancing group performance and honor, a knowledge-sharing culture exists, and relational values mitigate competitive interpretations, we proposed the following hypotheses:

**Hypothesis** **1.** *Innovative behavior negatively predicts ostracism.*

### 2.2. Mediation Effect of Envy

The empirical evidence of the direct link between innovative behavior and ostracism is mixed and inconsistent. Some studies suggest that innovative employees, by deviating from group norms or achieving visible recognition, may provoke subtle forms of exclusion from their peers (e.g., Mao et al., 2021; Breidenthal et al., 2020). However, other studies have failed to find a consistent or significant relationship, indicating that innovation does not automatically lead to ostracism (e.g., Wang et al., 2022). These inconsistencies suggest that ostracism is not a direct or inevitable consequence of innovative behavior. Instead, a fine-grained theoretical mechanism is required to explain the conditions and processes through which innovation translates into ostracism.

We propose that envy is a critical emotional mediator that explains how innovative behavior induces exclusionary outcomes. Envy is a unique emotion that strongly influences behavior (Smith & Kim, 2007). It is not merely a dispositional trait, but rather a relationally induced, situationally specific emotion (Parrott & Smith, 1993). From this perspective, the perception of being the target of envy—or, conversely, experiencing envy toward others—functions as an emotional black box that transforms social comparison into behavioral responses.

Drawing on affective events theory (Weiss & Cropanzano, 1996), we argue that innovative behavior constitutes a workplace event whose meaning depends on social appraisal. Innovation may represent a positive event, signaling creativity and potential improvements in performance. However, for peers, the same event may be appraised as a negative affective event because it highlights upward comparisons and evokes feelings of inferiority or threat. The most immediate and powerful emotional response in such situations is envy, which motivates defensive reactions such as ostracism. Thus, ostracism does not stem directly from innovation itself, but rather from the negative emotional process triggered by envy. This logic also helps reconcile the mixed empirical findings on the innovation–ostracism link in prior research.

Social exchange theory (Blau, 1964) explains that the consequences of envy are contingent on the quality of relational resources within an organization. When relational capital is scarce, innovative behavior heightens social comparison and intensifies envy, serving as an emotional bridge that leads to exclusion. Conversely, when relational resources are abundant, envy as a negative emotional pathway is weakened and innovative behavior may be reframed as creating opportunities for gratitude, stimulation, and learning (Duffy et al., 2012; Hargadon & Bechky, 2006). Therefore, the relational context moderates whether envy amplifies or attenuates the innovation–ostracism relationship.

In summary, envy represents the critical emotional mechanism through which innovative behavior translates into ostracism. Rather than being a direct and inevitable consequence, ostracism occurs specifically through envy, whose intensity varies depending on the relational context.

**Hypothesis** **2.** *Envy mediates the relationship between innovative behavior and ostracism. Specifically, envy constitutes an emotional black box that explains how innovation translates into exclusionary reactions.*

### 2.3. Moderating Mechanism of Frequency of Supervisor Interaction

Social comparison theory suggests that individuals evaluate their abilities and performance by comparing themselves with others (Festinger, 1954). However, the results of such comparisons are not always negative and may vary depending on how the behavior of the comparison target is interpreted. We propose that the frequency of supervisor interaction is a key factor that moderates the impact of innovative behavior on ostracism through envy.

Leader-member exchange theory (LMX; Graen & Uhl-Bien, 1995) explains that high-quality relationships with supervisors enhance member performance and attitudes. However, the effects of LMX extend beyond simple resource exchange. Frequent supervisor interactions fundamentally change how members’ behaviors are interpreted within an organization. According to the sense giving concept presented by Maitlis (2005), leaders play an active role in constructing the meaning of events and behaviors within organizations. Supervisor interaction plays an important role in shaping colleagues’ perceptions of innovative behavior. Frequent interactions clarify that innovative behavior contributes to the achievement of overall organizational goals rather than to personal gains.

Social cognitive theory (Bandura, 1977) emphasizes the importance of observers’ cognitive abilities in social learning processes. When innovative behavior appears complex and sometimes conflicts with existing practices, how colleagues interpret and respond depends largely on the interpretive framework provided by supervisors. The level of supervisor interaction determines the clarity and persuasiveness of the interpretive framework.

When the frequency of supervisor interaction is low, the intentions and value of innovative behavior remain ambiguous. Colleagues are likely to perceive innovative individuals’ behavior as a threat to themselves, triggering envy. This ambiguity is further exacerbated in collectivistic settings, where individual actions not clearly aligned with group goals can be readily interpreted as disrupting relational stability and challenging established norms, thus heightening the potential for negative social comparison and envy. Conversely, in situations where the frequency of supervisor interaction is high, innovative behavior is clearly recognized as valuable behavior that contributes to the team and the entire organization. In this context, innovative behavior suppresses envy and triggers the motivation for learning and cooperation. A high frequency of supervisor interaction, particularly in hierarchical Asian contexts, provides legitimate endorsement, framing innovative acts as aligned with collective progress and demonstrating that the supervisor has given face to the innovator, thereby minimizing perceived threats to group harmony and individual standing.

In summary, we propose that the frequency of supervisor interaction moderates the impact of innovative behavior on ostracism through envy. A high frequency of supervisor interaction ensures that innovative behavior is perceived as valuable behavior that contributes to the group, strengthening the envy-suppressing effect of innovative behavior. This is particularly important when innovative behavior appears complex and sometimes challenges existing practices.

Conversely, when the frequency of supervisor interaction is low, the intentions and value of innovative behavior are unclear, making colleagues likely to perceive it as threatening. In such situations, the effect of innovative behavior on reducing envy weakens; consequently, the effect of reducing ostracism will not appear. Therefore, we propose the following hypothesis:

**Hypothesis** **3.** *The frequency of supervisor interaction strengthens the indirect negative effect of innovative behavior on ostracism through envy. That is, when supervisor interaction is frequent and of high quality, innovative behavior is more strongly associated with reduced ostracism via lower envy.*

**Figure 1:**
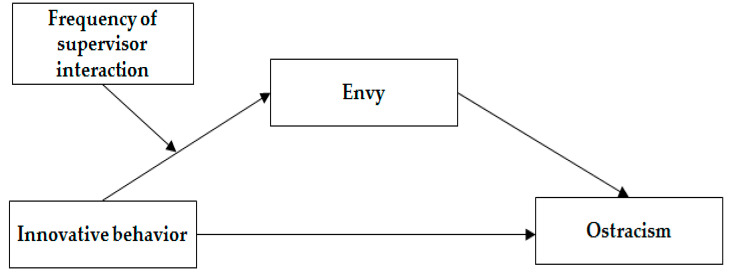
The research model.

The research model based on the above hypotheses is depicted in Figure 1.

## 3. Methods

### 3.1. Sample and Data Collection

This study utilized a two-wave longitudinal design to mitigate common method bias (CMB). Data were collected from 392 full-time employees across 20 South Korean private companies via Macromill Embrain, a reputable online survey platform with over 6.4 million verified panelists in Korea.

At Time 1, innovative behavior and frequency of supervisor interaction were measured. At Time 2 (1 month later), envy and workplace ostracism were assessed. Of the 608 initial respondents at Time 1, 489 completed the assessment at Time 2. After excluding incomplete or inconsistent responses, 392 valid responses were retained for the final analysis.

Of the 392 participants, 201 were male (51.3%) and 191 were female (48.7%). The participants’ ages were distributed as follows: 18.6% were in their 20s, 42.6% in their 30s, 28.3% in their 40s, 9.2% in their 50s, and 1.3% in their 60s. Regarding organizational tenure, 9.7% had less than 1 year of experience, 42.3% had between 1 and 5 years, 25.0% had between 5 and 10 years, 13.3% had between 10 and 15 years, 6.1% had between 15 and 20 years, and 3.6% had over 20 years of experience.

### 3.2. Measures

The constructs employed in this study were derived from previously validated scales with established reliability and validity, and were subsequently adapted to align with Korean cultural and research contexts.

#### 3.2.1. Innovative Behavior

Innovative behavior was assessed using the 3-item scale developed by Scott and Bruce (1994). A sample item is, “I seek out new methods or techniques when performing my work.” Participants responded using a 7-point Likert-type scale ranging from 1 (strongly disagree) to 7 (strongly agree), with higher scores indicating greater levels of self-reported innovative behavior. The innovative behavior scale demonstrated good internal consistency and reliability, with a Cronbach’s alpha coefficient of 0.880.

#### 3.2.2. Envy

Envy was measured using the 9-item scale developed by Cohen-Charash (2009). A sample item from the scale is “I feel envious of a certain colleague.” Participants rated their responses on a 7-point Likert scale ranging from 1 (strongly disagree) to 7 (strongly agree), with higher scores indicating greater levels of envy experienced in organizational settings. In the present study, the scale showed excellent internal consistency and reliability, with a Cronbach’s alpha value of 0.926.

#### 3.2.3. Workplace Ostracism

Workplace ostracism was measured using the 4-item scale developed by Lee et al. (2019). A sample item is, “Others in my organization ignore or dismiss my opinion.” Participants responded using a 7-point Likert scale ranging from 1 (strongly disagree) to 7 (strongly agree), with higher scores indicating greater perceived experiences of ostracism. In this study, this measure demonstrated excellent internal consistency and reliability with a Cronbach’s alpha value of 0.958.

#### 3.2.4. Frequency of Supervisor Interaction

The frequency of supervisor interaction was measured using a single-item scale adapted from Chun et al. (2009). This item assesses the frequency of direct interaction between the employee and their immediate supervisor, capturing the extent of their relational contact in the workplace. The item asked, “Looking back on the past 3 months, approximately how many hours per week do you spend interacting with the leader at work?” Participants responded by indicating the estimated number of hours per week, which was treated as a continuous variable in the analysis.

#### 3.2.5. Control Variables

In this study, gender, age, and organizational tenure were included as control variables to account for potential confounding effects on the relationships among the key constructs: innovative behavior, envy, ostracism, and frequency of supervisor interaction. Previous research suggests that gender can influence coworkers’ feelings of envy toward innovative behavior, with empirical evidence indicating that female innovators are more likely to elicit higher levels of envy among their peers (Breidenthal et al., 2020). Furthermore, older employees may be less receptive to innovative behavior because of their stronger attachment to established routines, which, in turn, may increase the likelihood of envy and ostracism among coworkers, as demonstrated in a meta-analysis. Organizational tenure is also closely linked to the accumulation of trust networks and social capital in the workplace. Consequently, innovative behavior by long-tenured employees is generally less likely to be perceived negatively by their colleagues. Based on these findings, this study adjusted for gender, age, and organizational tenure to examine the causal relationships among the primary study variables more accurately.

### 3.3. Analysis Strategy

Statistical analyses were conducted using SPSS 20 and AMOS 20 software. Confirmatory factor analysis (CFA) and reliability analysis (Cronbach’s alpha) were performed to assess the validity and reliability of the instruments. Descriptive statistics and Pearson’s correlation analyses were used to examine the characteristics of the variables and their relationships. The PROCESS macro in SPSS was used to test the hypotheses. Model 4 of the PROCESS macro was used to examine the mediating effect of envy on the relationship between innovative behavior and workplace ostracism. Model 7 was employed to test whether this mediating effect was moderated by the frequency of supervisor interaction, by assessing a moderated mediation effect. All indirect effects were tested using bootstrapping procedures with 10,000 resamples, and significance was determined using a 95% confidence interval (CI). Statistical significance was set at *p* < 0.05.

## 4. Results

### 4.1. Descriptive Statistics, Correlations

Descriptive statistics and Pearson’s correlations were obtained before hypothesis testing. Table 1 presents the means, standard deviations, and correlations of all the variables included in the study. Innovative behavior (M = 3.335, SD = 0.737) was negatively correlated with envy (r = −0.124, *p* < 0.05) and workplace ostracism (r = −0.158, *p* < 0.01), and positively correlated with frequency of supervisor interaction (r = 0.155, *p* < 0.01). Envy (M = 2.647, SD = 0.849) showed a significant positive correlation with workplace ostracism (r = 0.418, *p* < 0.001), but was not significantly correlated with frequency of supervisor interaction (r = −0.039, ns). Frequency of supervisor interaction (M = 5.798, SD = 4.370) was negatively correlated with workplace ostracism (r = −0.135, *p* < 0.01). All correlation coefficients were below the threshold of 0.80, indicating that multicollinearity was not a concern in this study (Gunst & Mason, 1980).

### 4.2. Reliability and Validity

To ensure the psychometric quality of the measurement scales, confirmatory factor analysis (CFA) was conducted to assess the fit and validity of the measurement model. The hypothesized four-factor model (innovative behavior, envy, ostracism, frequency of supervisor interaction) demonstrated acceptable fit (χ^2^ = 322.807, *df* = 93, TLI = 0.945, CFI = 0.957, and RMSEA = 0.080), meeting the recommended thresholds of TLI/CFI ≥ 0.90 and RMSEA ≤ 0.08 (Hu & Bentler, 1999). Composite reliability (CR) values ranged from 0.898 to 0.954 (all > 0.70), and average variance extracted (AVE) values ranged from 0.503 to 0.838 (all > 0.50), confirming strong convergent validity. Discriminant validity was established as the square root of AVE for each construct exceeded its correlations with other constructs, and all AVE values (0.503–0.838) were larger than the squared correlation coefficients (0.001–0.174) between latent variables (Fornell & Larcker, 1981). These results demonstrate that all of the measurement scales possessed adequate reliability and validity for hypothesis testing. The detailed CFA results and reliability analyses are summarized in Table 2.

### 4.3. Mediation Effect of Envy

The results of the mediation analysis examining the role of envy in the relationship between innovative behavior and ostracism are presented in Table 3. The total effect of innovative behavior on ostracism was significant (B = −0.229, t = −3.381, *p* < 0.01). Innovative behavior had a significant negative effect on envy (B = −0.153, t = −2.585, *p* < 0.05) and had a significant direct negative effect on ostracism (B = −0.160, t = −2.547, *p* < 0.05). Additionally, the detailed results of the mediation test are presented in Table 4. Envy significantly and positively predicted ostracism (B = 0.450, t = 8.404, *p* < 0.001). To test for indirect effects, a bootstrapping procedure with 10,000 resamples was used to generate a 95% bias-corrected confidence interval. The indirect effect of innovative behavior on ostracism via envy was significant (B = −0.069). The confidence interval did not include zero (95% CI [−0.131, −0.013]), indicating a significant mediating effect of envy. As the direct effect remained significant (B = −0.160, *p* < 0.05), envy partially mediated the relationship, supporting Hypothesis 2.

### 4.4. Moderated Mediation of Frequency of Supervisor Interaction

Before testing the moderated mediation effect, we verified the moderating role of the frequency of supervisor interaction in the relationship between innovative behavior and envy. As shown in Table 5, The interaction term (innovative behavior × frequency of supervisor interaction) significantly predicted envy (B = −0.034, SE = 0.012, *p* < 0.05), indicating that a higher frequency of supervisor interaction strengthened the negative effect of innovative behavior on envy.

**Figure 2:**
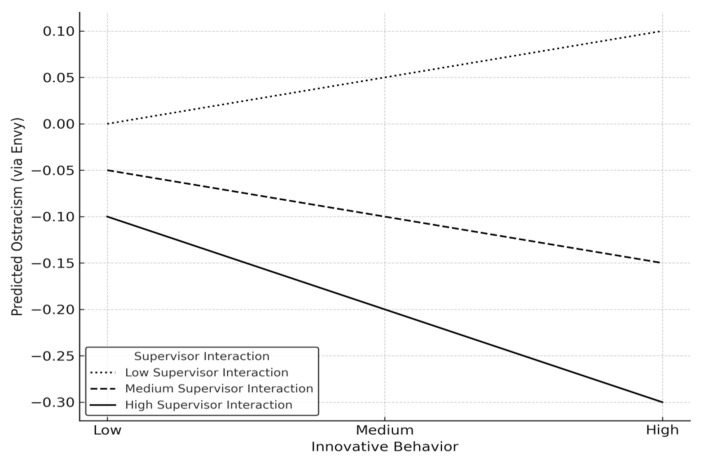
Moderation effect.

To test the moderated mediation hypothesis (H3), moderated mediation analysis was conducted using PROCESS Model 7. As shown in Table 6, the index of moderated mediation was significant, Index = −0.015, SE = 0.006, 95% CI [−0.028, −0.004], indicating that the indirect effect of innovative behavior on ostracism through envy varied significantly depending on the frequency of supervisor interaction. The confidence intervals for the mean and high interaction levels did not include zero, thus providing evidence of significant conditional indirect efforts. This result suggests that the negative indirect effect (innovative behavior, reduced envy, and reduced ostracism) became stronger when the frequency of supervisor interaction was high (Figure 2).

## 5. Discussion

This study analyzed the impact of innovative behavior on envy and ostracism, and supported a moderated mediation effect of the frequency of supervisor interaction in this relationship. Based on longitudinal study results from 392 South Korean corporate employees, innovative behavior appeared to reduce envy (β = −0.153, *p* < 0.05) and thereby reduced ostracism (indirect effect = −0.069, 95% CI [−0.131, −0.013]). Notably, the indirect effect varied depending on the frequency of supervisor interaction. When frequency of supervisor interaction was high, the envy-reducing effect of innovative behavior was strengthened, leading to a significant reduction in ostracism (β = −0.127, 95% CI [−0.214, −0.054]). In contrast, when frequency of supervisor interaction was low, this effect did not emerge (β = 0.007, ns). These results suggest that the frequency of supervisor interaction functions not merely as a buffer that mitigates negative effects but also as a factor that activates positive effects under high levels of interaction. That is, when supervisor–subordinate interaction is sufficiently strong, innovative behavior is more likely to translate into positive outcomes through a reduction in envy.

Our findings have two important implications. First, the social consequences of innovative behavior are not universal and can fundamentally vary according to the organizational context. Second, the frequency of supervisor interaction appears to be a key mechanism that reconstructs the meaning of innovative behavior, transforming negative outcomes into positive ones. This suggests that the dark side of creativity is not inevitable but rather a manageable conditional phenomenon.

### 5.1. Theoretical Implications

This study makes the following theoretical contributions to the creativity and innovation literature. First, this study strongly suggests that organizations should actively manage the social consequences of innovative employee behavior and not merely promote it. Our findings, particularly the pivotal role of frequency of supervisor interaction in buffering the links among innovative behavior, envy, and ostracism, offer several key practical implications.

While innovative behavior is a clear organizational asset, it carries the social risk of inducing envy and ostracism among coworkers. Our research highlights supervisors as the central actors in mitigating this risk. Organizations cannot simply encourage innovation in a vacuum; rather, they must cultivate a relational environment in which innovation is perceived as positive rather than a social threat.

Based on our findings, we propose the following practical strategies to maximize the positive effects of innovative behavior and mitigate its negative effects. First, conscious managerial intervention is likely crucial. Supervisors must intervene deliberately to ensure that innovative employees do not become targets of coworker envy or ostracism. This could likely be achieved by explicitly framing innovative performance not as an individual’s exclusive achievement but rather as a behavior aligned with team and organizational goals.

Second, enhancing relational communication also appears essential. Through regular team meetings or one-on-one sessions, supervisors should consistently communicate the positive value of innovation. This form of interaction could help reframe innovation from a threatening source of uncertainty into a learning opportunity for the entire team, thereby transforming envy into constructive motivation or a drive for learning.

Third, building a social monitoring system may also be vital. Managers must be sensitive to emotional and relational dynamics within their teams. Using informal conversations and close observation, they could identify subtle signs of envy or ostracism early and promptly intervene to mediate conflict. This strategy emphasizes the importance of relational competence in leadership training programs.

In conclusion, our study suggests that managing the dark side of innovation requires managers to engage actively. By proactively managing the social implications of innovative behavior, organizations can increase the chance of simultaneously achieving innovation and relational harmony within teams. This provides a crucial strategic insight for successfully managing innovation in today’s complex organizational landscape.

### 5.2. Limitations and Future Research

The present study offers significant theoretical and practical implications but also has several limitations. A primary limitation is the measurement of the key variables. Specifically, the moderator, namely the frequency of supervisor interaction, was measured using a single item that captured only its frequency and not the quality central to LMX theory. This creates a clear discrepancy between our theoretical concepts (e.g., sense giving and value clarification) and our empirical measurements. Future research should employ more robust multi-item scales that include the qualitative dimension of interaction (e.g., the LMX-7 scale) to validate our moderation hypothesis more precisely.

Second, data were collected from 20 companies, providing a nested data structure at the organizational level. This means that organizational-level variables such as corporate culture, policies, and leadership styles could act as potential confounding factors that influence relationships at the individual level. As this study focused on individual-level analyses, we could not explicitly adjust for these effects. Therefore, the findings should be interpreted without considering specific organizational contexts. Future research should use appropriate statistical methods, such as multilevel modeling, to account for the nested data structure and explore how organizational-level variables influence relationships at the individual level.

Third, the reliance on self-reported survey data raises concerns about common methods and social desirability biases. Since sensitive topics such as envy and ostracism were investigated, it is possible that the respondents underreported their experiences. To enhance methodological robustness, future research should incorporate data from multiple sources such as peer ratings and supervisor reports to measure these variables.

Fourth, this study had measurement limitations. While our hypotheses were premised on situations in which coworkers envy the focal employee, the actual survey items were designed to capture individuals’ reports of their own envy of their colleagues. This discrepancy may have created misalignment between the measurement and theoretical framework; thus, caution is warranted when interpreting the findings. Future research should distinguish between others’ envy toward the focal individual and the focal individual’s envy toward others and adopt peer reports or multi-source data collection to more accurately capture the inherently interactional nature of envy.

Fifth, this study collected data from 20 different companies, which implies that organizational-level factors, such as corporate culture, organizational policies, and leadership styles, may have influenced the results. However, because our focus was on individual-level relationships, we did not adjust for organizational-level effects. Therefore, the findings should be interpreted without considering the organizational context. Future research should employ appropriate analytical techniques such as multilevel modeling to more rigorously account for and examine organizational-level influences.

Finally, the generalizability of the study is limited, as the sample was collected exclusively from employees of South Korean companies. The unique cultural characteristics of Korea, marked by collectivism and relationship-oriented contexts, may have influenced the results. The impact of innovative behavior on envy and ostracism may differ across cultures. Future comparative studies between individualistic (e.g., the United States and Western Europe) and collectivistic cultures (e.g., Japan and China) are needed to uncover how cultural differences shape the social effects of innovative behavior. Moreover, studies should explore how these effects vary based on organizational size, industry characteristics, and innovation intensity. Such investigations will contribute to a more comprehensive and nuanced understanding of the social consequences of innovative behavior, and offer practical guidelines for organizations seeking to foster innovation while maintaining harmonious interpersonal relationships.

## Figures and Tables

**Table 1:** Descriptive statistics and correlations.

Variables	Mean	SD	1	2	3
1. Innovative behavior	3.335	0.737			
2. Envy	2.647	0.849	−0.124 *		
3. Ostracism	1.924	0.981	−0.158 **	0.418 ***	
4. Frequency of supervisor interaction	5.798	4.370	0.155 **	−0.039	−0.135 **

Note: * *p* < 0.05, ** *p* < 0.01, *** *p* < 0.001.

**Table 2:** Results of confirmatory factor analysis for the measurement model.

	λ	S.E.	CR	AVE
Innovative behavior			0.913	0.779
I seek out new methods or technologies when doing my work	0.776			
I try to incorporate new ideas into my work	0.890	0.067		
I make efforts to turn innovative ideas into practical changes	0.862	0.065		
Envy			0.898	0.503
I dislike a certain colleague	0.886			
I have negative feelings toward a certain colleague	0.916	0.029		
I harbor ill will toward a certain colleague	0.773	0.034		
I have mixed feelings (both positive and negative) toward a certain colleague	0.694	0.031		
I feel irritated by a certain colleague	0.840	0.043		
I want something that a certain colleague has	0.604	0.047		
I feel inferior to a certain colleague	0.572	0.048		
A certain colleague seems to have more than I do	0.592	0.048		
I feel envious of a certain colleague	0.555	0.048		
Workplace Ostracism			0.954	0.838
treat me as if I am invisible	0.921			
ignore my opinion	0.953	0.029		
exclude me from their gathering	0.890	0.034		
avoid interacting with me	0.925	0.031		

χ^2^ = 322.807, *df* = 93, TLI = 0.945, CFI = 0.957, RMSEA = 0.080.

**Table 3:** Mediation effect test.

	Dependent Variable	Independent Variable	Coefficient	SE	t	LLCI	ULCI
Step1	Envy	Innovative behavior	−0.153	0.059	−2.585 *	−0.270	−0.037
		Gender	−0.255	0.091	−2.806 *	−0.434	−0.076
		Age	−0.008	0.006	−1.348	−0.019	0.004
		organizational tenure	0.018	0.040	0.454	−0.060	0.096
Step2	Ostracism	Innovative behavior	−0.160	0.063	−2.547 *	−0.284	−0.037
		Envy	0.450	0.053	8.404 ***	0.344	0.555
		Gender	−0.252	0.097	−2.610 **	−0.442	−0.062
		Age	−0.002	0.006	−0.264	−0.014	0.010
		Organizational tenure	−0.056	0.042	−1.335	−0.138	0.026

Model summary for Step1: R^2^ = 0.036, F = 3.634 **. Model summary for Step2: R^2^ = 0.203, F = 19.667 ***. Note: Gender was dummy coded (0 = male, 1 = female). * *p* < 0.05, ** *p* < 0.01, *** *p* < 0.001.

**Table 4:** Effect of variables (mediation).

**Direct Effect of Innovative Behavior (X) on Ostracism (Y)**				
**Effect**	**SE**	**t**	**LLCI**	**ULCI**
−0.160	0.063	−2.547 *	−0.284	−0.037
**Indirect Effect of Innovative Behavior (X) on Ostracism (Y)**				
	**Effect**	**Boot SE**	**LLCI**	**ULCI**
Envy(M)	−0.069	0.030	−0.131	−0.013

Note: * *p* < 0.05.

**Table 5:** Moderation effect test.

	Dependent Variable	Independent Variable	Coefficient	SE	t	LLCI	ULCI
Step1	Envy	Innovative behavior	−0.134	0.060	−2.222 *	−0.253	−0.015
		Frequency of supervisor interaction	0.000	0.010	0.036	−0.019	0.020
		Interaction	−0.034	0.012	−2.801 **	−0.058	−0.010
		Gender	−0.269	0.091	−2.974 **	−0.447	−0.091
		Age	−0.009	0.006	−1.530	−0.021	0.003
		Organizational tenure	0.020	0.039	0.501	−0.058	0.097
Step2	Ostracism	Innovative behavior	−0.160	0.063	−2.547 *	−0.284	−0.037
		Envy	0.450	0.053	8.404 ***	0.344	0.555
		Gender	−0.252	0.097	−2.610 **	−0.442	−0.062
		Age	−0.002	0.006	−0.264	−0.014	0.010
		Organizational tenure	−0.056	0.042	−1.335	−0.138	0.026

Model summary for Step1: R^2^ = 0.056, F = 3.783 **. Model summary for Step2: R^2^ = 0.203, F = 19.667 ***. Note: Gender was dummy coded (0 = male, 1 = female). * *p* < 0.05, ** *p* < 0.01, *** *p* < 0.001.

**Table 6:** Moderated mediation effect test.

**Index of Moderation Mediation**				
	**Index**	**Boot SE**	**Boot LLCI**	**Boot ULCI**
Frequency of supervisor interaction (M)	−0.015	0.006	−0.029	−0.004
**Indirect Effect**				
	**Index**	**Boot SE**	**Boot LLCI**	**Boot ULCI**
−1 SD score (Low)	0.007	0.040	−0.069	0.089
Mean score (Medium)	−0.060	0.029	−0.120	−0.005
+1 SD score (High)	−0.127	0.041	−0.214	−0.054

## Data Availability

Data supporting the reported results are available from the authors upon request.
